# The 2014 Ming K Jeang Award for Excellence in *Cell & Bioscience*

**DOI:** 10.1186/s13578-015-0014-1

**Published:** 2015-05-18

**Authors:** Yun-Bo Shi

**Affiliations:** The National Institutes of Health, Bethesda, MD USA

## Abstract

Three research groups led by Dr. Robert Clarke of Georgetown University Medical Center, Washington, DC, USA; Dr. Lixin Wei of Shanghai Jiaotong University, Shanghai, China; and Dr. Zhiming Zhang of Xiamen University, Xiamen, Fujian, China, won the 2014 Ming K Jeang Award for Excellence in *Cell & Bioscience.*

## Editorial

We are very pleased to announce that three research groups, who each published an outstanding research article in *Cell & Bioscience* in 2014, have been selected to receive the Ming K Jeang Award for Excellence in *Cell & Bioscience*. The Ming K Jeang Award for Excellence in *Cell & Bioscience* was established in 2011 with a generous donation from the Ming K. Jeang Foundation to honor outstanding research articles published in *Cell & Bioscience*, the official journal of the Society of Chinese Bioscientists in America (SCBA; www.scbasociety.org). A committee of *Cell & Bioscience* Editors, chaired by Dr. Dong-Yan Jin, considered all research articles published in the journal in 2014 to select the following three articles to receive the award [1–3]:

- Mitochondria directly donate their membrane to form autophagosomes during a novel mechanism of parkin-associated mitophagy
  Katherine L Cook, David R Soto-Pantoja, Mones Abu-Asab, Pamela AG Clarke, David D Roberts, Robert Clarke *Cell & Bioscience* 2014, **4**:16 (27 March 2014)
  Abstract | Full text | PDF | ePUB | PubMed | Cited on BioMed Central
- Autophagy protects against palmitate-induced apoptosis in hepatocytes
  Ning Cai, Xue Zhao, Yingying Jing, Kai Sun, Shufan Jiao, Xiaojing Chen, Haozheng Yang, Yan Zhou, Lixin Wei *Cell & Bioscience* 2014, **4**:28 (21 May 2014)
  Abstract | Full text | PDF | ePUB | PubMed
- MicroRNA-20b promotes cell growth of breast cancer cells partly via targeting phosphatase and tensin homologue (PTEN)
  Weidong Zhou, Guixiu Shi, Qiuyan Zhang, Qiuwan Wu, Boan Li, Zhiming Zhang *Cell & Bioscience* 2014, **4**:62 (14 October 2014)
  Abstract | Full text | PDF | ePUB | PubMed

Congratulations to these three groups of investigators for jobs well done!

We are looking forward to receiving contributions of outstanding research articles from the scientific community in 2015 and beyond.
